# Therapeutic Observations upon Carbo Vegetabilis

**Published:** 1889-06

**Authors:** C. Hering


### THERAPEUTIC OBSERVATIONS UPON CARBO VEGETABILIS.

Carbo vegetabilis, though so often applicable in the abuse of Quinine, is still more so as an antidote to the injurious effects of Calomel, particularly for the sensitiveness to every change of weather by which its use is so frequently attended. A high state of atmospheric temperature often causes nausea and sickness of the stomach, which are greatly aggravated by partaking of the tepid or otherwise bad water of large cities. These symptoms (very often to be met with in persons who, by their profession, are exposed to the severest heat of the day) have been frequently relieved by Carbo vegetabilis, even in some cases where, by the palliating, but too sudden cooling effect of ice-water, asthenic fever had been induced. The latter was sometimes accompanied by diarrhœa, and in such cases Bryonia was administered, either previously, or subsequently to Carbo veg. It appears singular that Carbonic acid, so well known for its refreshing coolness, was of no avail in these affections. A third equally new observation we owe to one of the most zealous friends and advocates of Homœopathy, who, for want of a physician, was compelled to attend his own child. It was but a few weeks old, when, in spite of the utmost attention to cleanliness, it became excoriated to such a degree, that the epidermis was destroyed, not only at the usual places, but also behind the ears and about the neck, presenting raw surfaces of considerable extent. Sulphur and Lycopodium relieved the little sufferer in some measure, but Carbo veg. very soon effectually cured him. We have since had occasion to observe this effect of Carbo veg. in several other instances.

During the autumn of 1833, the whooping-cough prevailed epidemically in Philadelphia, and the usual remedies, such as Drosera, Cina, Veratrum, and Sulphur, had but little effect in relieving the paroxysms, which generally ended with vomiting; but upon administering Carbo veg. the disease soon yielded. The same beneficial effect was experienced by other practitioners in 1836, and we likewise found that in the catarrhal stage of the disease, as well as for its sequelæ, it could also be relied on. In catarrhs attended with a characteristic hoarseness in the morning or at night, Carbo veg. is often beneficial. The influenza in the autumn of 1834 generally yielded either to Hepar sulph. or to Merc. viv.; but when the hoarseness just mentioned remained or recurred after a new cold, it was removed by Carbo veg. We also succeeded in curing a considerable number of cases of "the mumps" with Carbo veg., though Merc. solub. is the usual remedy for that disease.

This observation, in conjunction with the one mentioned at the beginning of these remarks, indicate an affinity between Mercury and Carbo veg., whilst the curative effects of the latter in removing the injurious consequences of ice water appear, moreover, to confirm its affinity to Arsenic, which is already established by the fact that both remedies are often employed in intermitting fevers, and that they have characteristic burning pains, offensive and easily bleeding ulcers, and many other symptoms in common. When Carbo veg. thus appears to rank between two such different metals, it follows that its affinity with the two relates to different spheres of action, as we may see by the diagnostics of these remedies. Lachesis is one of the antidotes of Carbo veg., either when the latter has been taken in its crude state, or homœopathically prepared, particularly when its effects are manifested by soreness of the gums, mouth, or throat.

From the effects of Carbo veg., we also see demonstrated the important truth that the pathogenetic and the therapeutic effects of medicinal agents perfectly correspond with their chemical action, which we see also exemplified in the effects of Arsenic, Causticum, Kreosote, Cantharides, and Lachesis, which remove symptoms similar to those produced by the bite of a snake. Dr. Franz, in treating of Ranunculus bulbosus, also remarks that the local symptoms occasioned by the external application, and those proceeding from its internal use are identical, and Y—— makes the same remark in regard to Ranunculus sceleratus. In order to become duly impressed with the practical import of this proposition, we ought to consider it in its connection with other doctrines and demonstrate its relation thereto, which our space will not permit us to do on the present occasion. We, therefore, only remark that Hahnemannism, or the pathogenetic action of certain substances, bears the same relation to general organic action that electricity bears to magnetism. This proposition is of the same importance in Homœopathy that Oerstedt's electro-magnetism is in natural philosophy. C. HERING.

